# Effectiveness of inactivated and Ad5-nCoV COVID-19 vaccines against SARS-CoV-2 Omicron BA. 2 variant infection, severe illness, and death

**DOI:** 10.1186/s12916-022-02606-8

**Published:** 2022-10-20

**Authors:** Zhuoying Huang, Shuangfei Xu, Jiechen Liu, Linlin Wu, Jing Qiu, Nan Wang, Jia Ren, Zhi Li, Xiang Guo, Fangfang Tao, Jian Chen, Donglei Lu, Xiaodong Sun, Weibing Wang

**Affiliations:** 1 Institute of Immunization, Shanghai Municipal Center of Disease Control and Prevention, 1380 Western Zhongshan Road, Shanghai, 200336 China; 2 Shanghai Institute of Infectious Disease and Biosecurity, School of Public Health, Fudan University, 138 Yi Xue Yuan Road, Shanghai, 200032 China; 3 Institute of Infectious Diseases, Shanghai Municipal Center of Disease Control and Prevention, Shanghai, 200336 China; 4 Division of Health Risk Factors Monitoring and Control, Shanghai Municipal Center of Disease Control and Prevention, Shanghai, 200336 China; 5 Key Laboratory of Public Health Safety of Ministry of Education, Fudan University, Shanghai, 200032 China

**Keywords:** COVID-19, Vaccine effectiveness, Case-control study, Inactivated vaccine

## Abstract

**Background:**

Limited data are available on the effectiveness of inactivated and Ad5-nCoV COVID-19 vaccines in real-world use—especially against Omicron variants in SARS-CoV-2 infection-naïve population.

**Methods:**

A matched case-control study was conducted among people aged ≥ 3 years between 2 December 2021 and 13 May 2022. Cases were SARS-CoV-2-infected individuals, individuals with severe/critical COVID-19, or COVID-19-related deaths. Controls were selected from consecutively test-negative individuals at the same time as cases were diagnosed and were exact-matched on year-of-age, gender, birthplace, illness onset date, and residential district in ratios of 1:1 with infected individuals and 4:1 with severe/critical COVID-19 and COVID-19-related death. Additionally, two subsets were constructed to analyze separate vaccine effectiveness (VE) of inactivated vaccines (subset 1) and Ad5-vectored vaccine (subset 2) against each of the three outcomes.

**Results:**

Our study included 612,597 documented SARS-CoV-2 infections, among which 1485 progressed to severe or critical illness and 568 died. Administering COVID-19 vaccines provided limited protection against SARS-CoV-2 infection across all age groups (overall VE: 16.0%, 95% CI: 15.1–17.0%) but high protection against severe/critical illness (88.6%, 85.8–90.8%) and COVID-19-related death (91.6%, 86.8–94.6%). In subset 1, inactivated vaccine showed 16.3% (15.4–17.2%) effective against infection, 88.6% (85.8–90.9%) effective against severe/critical COVIID-19, and 91.7% (86.9–94.7%) against COVID-19 death. Booster vaccination with inactivated vaccines enhanced protection against severe COVID-19 (92.7%, 90.1–94.6%) and COVID-19 death (95.9%, 91.4–98.1%). Inactivated VE against infection began to wane 12 weeks after the last dose, but two and three doses sustained high protection levels (> 80%) against severe/critical illness and death, while subset 2 showed Ad5-vectored vaccine was 13.2% (10.9–15.5%) effective against infection and 77.9% (15.6–94.2%) effective against severe/critical COVIID-19.

**Conclusions:**

Our real-world study found high and durable two- and three-dose inactivated VE against Omicron-associated severe/critical illness and death across all age groups, but lower effectiveness against Omicron infection, which reinforces the critical importance of full-series vaccination and timely booster dose administration for all eligible individuals.

**Supplementary Information:**

The online version contains supplementary material available at 10.1186/s12916-022-02606-8.

## Research in context

### Evidence before this study

We searched medRxiv, PubMed, and SSRN on 26 May 2022, using the terms “omicron,” “inactivated vaccines,” “adenovirus vector,” and “vaccine effectiveness,” with no language restrictions. Studies in Israel, the UK, and the USA demonstrated that mRNA vaccines with booster doses protect against serious illness caused by SARS-CoV-2 and Omicron variants; studies have also shown reduced neutralizing antibody activity against Omicron and a waning of protection following mRNA vaccination. Fewer data are available on real-world vaccine effectiveness (VE) of inactivated and adenovirus type 5-vectored vaccines, especially against Omicron. Most published studies of inactivated or ad5-vectored vaccine focused on safety, immunogenicity, or efficacy and were conducted among narrowly defined populations such as healthcare workers in Brazil. A recent study in Hong Kong during Omicron outbreaks estimated VE of three doses of CoronaVac against mild/moderate disease to be 17.9% for adults aged 20–59 years and 50.7% for adults aged above 60 years; VEs against severe/fatal COVID-19 were above 95% across all age groups.

### Added value of this study

Our real-world study in a SARS-CoV-2 infection-naïve population showed that inactivated vaccines and Ad5-nCoV had high and durable VE against Omicron-associated severe/critical illness and death across all age groups but much lower effectiveness against Omicron infection. Booster doses offered additional protection against severe/critical disease and death with a duration of at least 36 weeks.

### Implications for all the available evidence

These findings provide strong evidence for completing full-series and booster vaccination among eligible, recommended populations in China. Although inactivated and Ad5-nCoV vaccines provided limited protection against Omicron infection, they were highly effective for preventing severe illness and death across all age groups—even higher with homologous boosting. Incorporation of our data on the risk of hospitalization and death could inform policy-making decisions on the speed, nature, and duration of public health and social interventions for mitigating the pandemic. All residents eligible to be vaccinated who are without medical contraindication should receive a full primary series; individuals eligible for booster doses should receive timely booster doses. Improving coverage among the elderly is especially important, as the elderly suffer most from COVID-19.

## Background

The Omicron BA. 2 severe acute respiratory syndrome coronavirus 2 (SARS-CoV-2) variant of concern was first detected in the USA in November of 2021 and has subsequently spread globally [1]. Sequencing of virus isolates from 129 coronavirus disease 2019 (COVID-19) patients between late February 2022 and May 2022 showed that Omicron BA. 2 was the dominant sub-lineage in Shanghai [2]. Omicron BA. 2 differs by approximately 40 mutations from the original Omicron lineage, BA. 1, and has a growth advantage over BA. 1 [3]. While studies are ongoing to understand the reasons for this growth advantage, initial data suggest that BA.2 appears more transmissible than BA. 1, which currently remains the most common Omicron sub-lineage reported. Rodent models suggest that the infectivity and pathogenicity of Omicron/BA.2 variant are similar to Omicron/BA. 1’s [4, 5].

Studies in Israel, the UK, and the USA have demonstrated that primary-series and booster vaccination with mRNA vaccines protect against SARS-CoV-2 infection, with greater protection from COVID-19-related hospitalizations and severe outcomes [6–8] which is also seen against Omicron outcomes [9–11]. Studies have also reported reduced neutralizing antibody activity against Omicron [12]. Fewer data are available on real-world vaccine effectiveness (VE) of inactivated and adenovirus type 5-vectored vaccine, especially against Omicron. A Singaporean study before Omicron became prevalent reported that compared with BNT162b2, subjects who received inactivated whole virus vaccines were more likely to be infected with SARS-CoV-2 [13]. An ecological VE study in Hong Kong found that three doses of either BNT162b2 or CoronaVac provided substantial protection against severe COVID-19 (VE > 95%) and death (VE > 96%) caused by confirmed Omicron variant infection [14].

In Shanghai, COVID-19 vaccination started in February 2021 among residents 18–59 years old, in March 2021 among residents 60–75 years of age, in May 2021 for individuals 76 years and older, and in September 2021 for children 3–17 years of age. Booster vaccination started in November 2021 for residents ≥ 18 years; children are not yet eligible for boosters. As of May 2022, there is no approved COVID-19 vaccine for children < 3 years in China. One out of every four doses of COVID-19 administered globally has been CoronaVac. Despite its global importance, limited evidence is available on the efficacy or effectiveness of this vaccine.

Between 2 December 2021 and 13 May 2022, a total of 618,019 individuals tested RT-PCR-positive for SARS-CoV-2 infection. By that time, over 90% of the population aged ≥ 3 years were reported to have received a primary series, and 47% received a booster dose. With its SARS-CoV-2 infection-naïve population, Shanghai provided a nearly unique outbreak setting to estimate real-world, vaccine-only-induced inactivated and Ad5-vectored COVID-19 VE against documented SARS-CoV-2 Omicron infection, severe or critical COVID-19, and COVID-19-related death.

## Methods

### Study setting and study participants

The study was conducted among people living in Shanghai, a provincial-level municipality in China with a resident population of more than 25 million people. Everyone living in Shanghai—citizens, foreigners, and immigrants—underwent several rounds of SARS-CoV-2 real-time polymerase chain reaction (RT-PCR) testing between 2 December 2021 and 13 May 2022. Complete demographic (age, gender, birthplace, and residential district), vaccination, and RT-PCR testing data were available to public health officials and study investigators. Individuals with negative RT-PCR SARS-CoV-2 tests who had tested positive before 2 December 2021 were excluded. Children less than 3 years of age were not vaccine-eligible and were excluded.

Controls were selected from consecutively test-negative individuals at the same time as cases were diagnosed. The size of the potential control group was 25.18 million people receiving several rounds of city-wide nucleic acid amplification testing (NAAT), and RT-PCR testing was universal regardless of COVID-19-associated symptoms.

### Study design

**Fig. 1:**
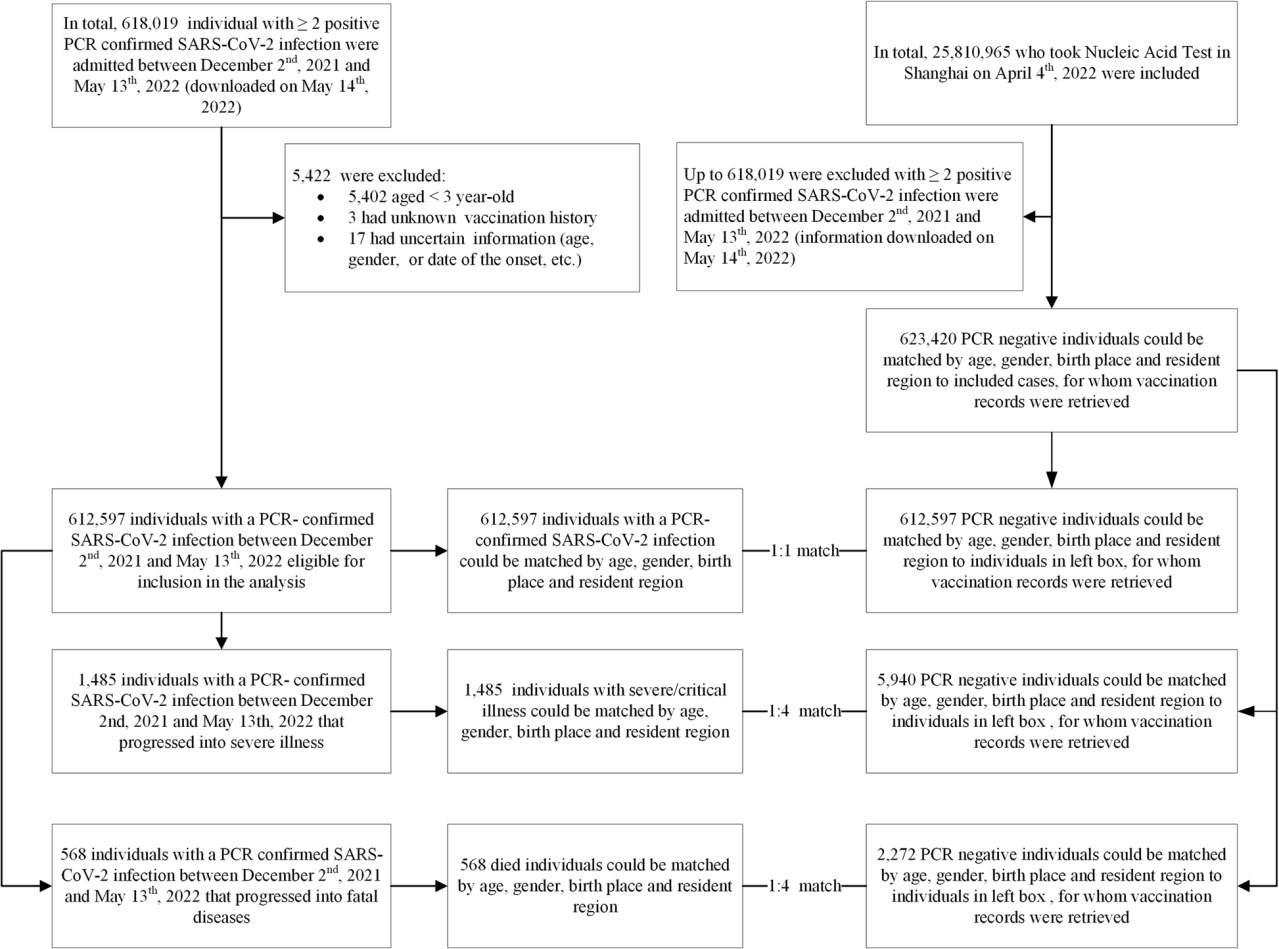
Participant enrollment flowchart

We used a matched case-control design for data extraction. Cases were individuals with documented SARS-CoV-2 infection, severe or critical COVID-19, or COVID-19-related death. Infection-only cases were matched 1:1 with controls on year-of-age, gender, birthplace (Shanghai/other places), date of testing, and residential district; severe or critical COVID-19 cases and COVID-19-related death cases were matched 1:4 on year-of-age, gender, birthplace, date of illness onset, and residential district (Fig. 1). We matched each case to unique controls without replacement. When there is more than one case-control pair available, we selected the first (for infection-only cases) or first four (for severe/critical and fatal cases) eligible individuals. We constructed two subsets to analyze separate VEs of inactivated vaccines (subset 1) and Ad5-vectored vaccine (subset 2) against each of the three outcomes (Additional file 1: Figs. S1 and S2).

### Infections and outcomes

PCR testing for SARS-CoV-2 is available in public hospitals and private laboratories throughout China. Since 10 March 2022, Shanghai conducted several rounds of SARS-CoV-2 tests, each involving more than 25 million people. The severity of disease for all SARS-CoV-2 infections was assessed in any of 48 COVID-19-designated hospitals in accordance with the *Diagnosis and Treatment Protocol for COVID-19 (trial version 9)* [15]. We included three outcomes in our study: documented SARS-CoV-2 infection, severe/critical COVID-19, and COVID-19-related death. Documented SARS-CoV-2 infection was confirmed by a positive RT-PCR test. For adult cases, severe illness must meet any of the following criteria: (a) respiratory distress (respiration rate [RR] ≥ 30 breaths per minute (BPM)), (b) oxygen saturation ≤ 93% at rest, and (c) arterial partial pressure of oxygen/fraction of inspired oxygen ≤ 300 mmHg. Additionally, cases with chest imaging that shows obvious lesion progression within 24–48 h > 50% shall be managed as severe illness. For child cases, severe illness must meet any of the following criteria: (a) persistent high fever over 3 days; (b) tachypnea (RR ≥ 60 BPM for infants aged below 2 months; RR ≥ 50 BPM for infants aged 2–12 months; RR ≥ 40 BPM for children aged 1–5 years, and RR ≥ 30 BPM for children > 5 years), independent of fever and crying; (c) oxygen saturation ≤ 93% on finger pulse oximeter taken at rest; (d) labored breathing (moaning, nasal fluttering, and infrasternal, supraclavicular, and intercostal retraction), cyanosis, and intermittent apnea; (e) lethargy and convulsion; and (f) difficulty feeding and signs of dehydration. Critical illness must meet any of the following criteria: (a) respiratory failure and requiring mechanical ventilation, (b) shock, and (c) other organ failures that require intensive care unit (ICU) care. COVID-19-related death is assessed by medical institutions.

Data on all confirmed and asymptomatic cases between 2 December 2021 and 13 May 2022 were obtained from the National Notifiable Diseases Registry System (NNDRS).

### Vaccination data

In this study, inactivated vaccine included Sinovac-CoronaVac, Sinopharm/BIBP COVID-19 vaccine, and Sinopharm/WIBP COVID-19 vaccine; Ad5-vectored vaccine referred to Cansino Ad5-nCoV-S COVID-19 vaccine; and recombined protein vaccine referred to recombinant SARS-CoV-2 vaccine (CHO cell), Anhui Zhifei Longcom Biopharmaceutical Institute of Microbiology.

The Shanghai Group Immunization System captures all vaccine administrations and is updated daily. This immunization information system is the repository of nearly all vaccinees’ records and includes the name, national identification number, vaccine type, vaccination date, vaccination dose, vaccination site, and vaccine manufacturer. This system is linked to the National Immunization Program Information System, which adds documented national identification-matching COVID-19 vaccinations received outside of Shanghai. Immunization histories were verified manually with records for subjects 3 years and older who had an unknown national identification number. NAAT data were linked to individual vaccination records using national identification numbers and names. Vaccination data were extracted and matched to cases and controls on 13 May 2022. Additional file 1: Fig. S3 shows the process of vaccination history retrieval.

Vaccination status was categorized into four levels in accordance with the national technical recommendations for COVID-19 vaccination: (1) unvaccinated—either no history of COVID-19 vaccination before the last SARS-CoV-2 exposure date; (2) partial vaccination—either one dose of inactivated vaccine, or two doses of inactivated vaccine but receiving the second dose within 14 days before the last SARS-CoV-2 exposure date, or two doses of recombinant protein vaccine (three doses are recommended for primary vaccination), or one dose of adenovirus vector vaccine or three doses of recombinant protein vaccine but with the most recent dose within 14 days before the last SARS-CoV-2 exposure date; (3) full primary vaccination—either two doses of inactivated vaccine, one dose of adenovirus vector vaccine, three doses of recombinant protein (CHO cell) vaccine with the most recent doses 14 days or more before the last SARS-CoV-2 exposure date and with no booster dose; or two doses of inactivated vaccine with one booster dose of inactivated vaccine, adenovirus vector vaccine, or recombinant protein (CHO cell) vaccine within 7 days before the last SARS-CoV-2 exposure date; or two doses of adenovirus vector vaccine within 7 days before the last exposure date; or (4) booster vaccination—either two doses of Ad5-vectored vaccine with the second dose 7 days or more before the last exposure date or two doses of inactivated vaccine and one booster dose of inactivated vaccine, adenovirus vector vaccine, or recombinant protein vaccine 7 days or more before the last exposure date.

Vaccination intervals were stratified by dose and interval (in weeks) post-vaccination at < 2, 3–12, 13–24, 25–36, and 37+ weeks after the first dose; < 2, 3–12, 13–24, 25–36, and 37+ weeks after the second dose; and < 2, 3–12, 13–24, and 25+ weeks after the third dose. Intervals (in days) were defined as the number of days between the onset date of infection and the date of the last vaccination minus 2 days, which was then converted into weeks. Detailed variable definitions are in Additional file 1: Table S1. Additionally, we performed a sensitivity analysis in which intervals (in days) were defined as the number of days between the testing positive date and the date of the last vaccination minus 7 days.

### Statistical analysis

Non-normally distributed continuous variables were expressed as medians (interquartile ranges (IQR)), and categorical variables were expressed as counts and proportions. Conditional logistic regression was used to estimate the odds ratio (OR) of vaccination among cases and controls, with documented SARS-CoV-2 infection, severe or critical COVID-19, and COVID-19-related death as dependent variables. Vaccination status was the independent variable, and VE was defined as 1 minus the matched OR. Vaccination status was defined using the date of onset and date of last vaccination, as above. Analyses were stratified by age category, gender, and vaccine type (inactivated vaccine, adenovirus vector vaccine, and recombinant protein vaccine). Matching was conducted with the use of JAVA, and analyses were performed with the use of RStudio 2022.02.3+492.

## Results

### Study population

There were 612,597 SARS-CoV-2 RT-PCR-confirmed cases in the study, among which 1485 progressed into severe or critical illness and 568 died (Table 1). The ratio of females to males for infection, severe/critical illness, and COVID-19 death were 0.8, 0.8, and 0.9, respectively. The most common age for infection was 40–59 years of age (37.3%); the most common age for severe/critical COVID-19 and COVID-19 death was 80 years and older (58.6% of severe/critical COVID-19 and 69.9% of COVID-19 deaths). Due to the exact matching, the demographic characteristics of controls and cases were the same (Table 1).

**Table 1:** Descriptive characteristics of confirmed COVID-19 cases in Shanghai between December 2, 2021, and May 13, 2022

Variable	SARS-CoV-2 infection (***n*** = 612,597)	Control 1 (***n*** = 612,597)	Severe or critical illness (***n*** = 1485)	Control 2 (***n*** = 5940)	Death (***n*** = 568)	Control 3 (***n*** = 2272)
**Age (median, IQR)**	45 (31, 58)	45 (31, 58)	83 (72, 89)	83 (72, 89)	86 (77, 90)	86 (77, 90)
**Age group (%)**						
3–17 years	35,553 (5.8)	35,553 (5.8)	1 (0.1)	4 (0.1)	-	-
18–39 years	216,769 (35.4)	216,769 (35.4)	13 (0.9)	52 (0.9)	2 (0.3)	8 (0.3)
40–59 years	228,387 (37.3)	228,387 (37.3)	95 (6.4)	380 (6.4)	22 (3.9)	88 (3.9)
60–79 years	112,635 (18.4)	112,635 (18.4)	506 (34.0)	2024 (34.0)	147 (25.9)	588 (25.9)
≥ 80 years	19,253 (3.1)	19,253 (3.1)	870 (58.6)	3480 (58.6)	397 ^a^(69.9)	1588 (69.9)
**Gender (%)**						
Female	273,152 (44.6)	273,152 (44.6)	654 (44.0)	2616 (44.0)	267 (47.0)	1068 (47.0)
Male	339,445 (56.4)	339,445 (56.4)	831 (56.0)	3324 (56.0)	301 (56.0)	1204 (56.0)
**Residential district (%)**						
Urban	225,697 (36.8)	225,697 (36.8)	816 (54.9)	3264 (54.9)	314 (55.3)	1256 (55.3)
Rural	386,900 (63.2)	386,900 (63.2)	669 (45.1)	2676 (45.1)	254 (44.7)	1016 (44.7)

^a^Two cases aged over 100 years old died and were matched to test-negative controls ± 5 years of age. Otherwise, due to exact matching, the demographic characteristics of controls and cases were the same

### Vaccination status

**Fig. 2:**
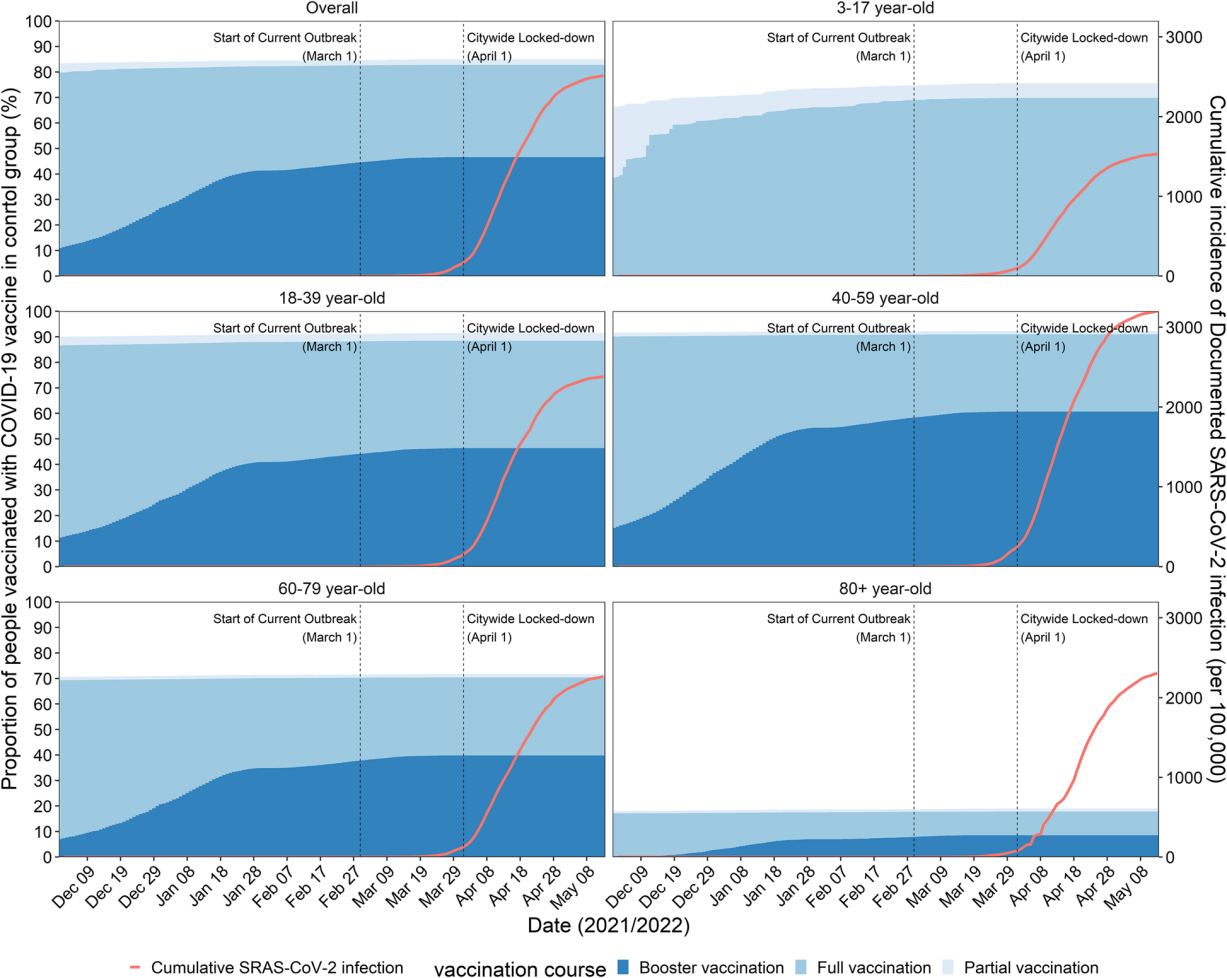
Vaccination status in the control group and cumulative documented SARS-CoV-2 infections by age grouping in Shanghai, 1 December 2021 through 13 May 2022. Also shown are public health and social measures implemented during the study period. Data for the cumulative incidence of SARS-CoV-2 infection was provided by Shanghai Health Commission

Among the male control group, 13.9% did not receive COVID-19 vaccine, 2.5% completed partial vaccination, 36.1% completed full vaccination, and 47.5% completed booster vaccination, while among the female control group, corresponding proportion was 19.2%, 2.0%, 35.6%, and 43.2%, respectively. Figure 2 shows the vaccination status of the control group over time and by age group. At the beginning of the study period, 82.7% of people received at least one dose of a COVID-19 vaccine, with the majority of 18–59 years old. The prevalence of the adult-focused booster dose increased from 11.9 to 49.0%. Full and partial coverage among adults aged 80 years and above was low and stable at approximately 15%. Figure 2 also shows the cumulative incidence of documented SARS-CoV-2 infection (250.9 per 10,000), of which the majority were 18–59 years old. Since March 12, a series of emergency epidemic prevention measures were implemented in Shanghai, including mass NAATs, citywide lockdowns, home quarantine of residents, isolation of cases, centralized quarantine of close contacts, and closed-loop management. The rapid spread of Omicron led to the temporary suspension of vaccination starting in March 2022.

### Vaccine effectiveness

COVID-19 vaccines provided some protection against SARS-CoV-2 infection in all age groups (overall VE: 16.0%, 95% CI: 15.1%, 17.0%), while in the age group of 40–59 years, protection was lower (VE: 7.5%, 95% CI: 5.5%, 9.5%). COVID-19 vaccines provided high effectiveness against severe/critical illness (VE: 88.6%, 95% CI: 85.8%, 90.8%) and COVID-19-related death (VE: 91.6%, 95% CI: 86.8%, 94.6%). Estimated VEs for full primary vaccination and booster doses against severe/critical illness were 83.8% (95% CI: 79.0%, 87.5%) and 92.8% (95% CI: 90.2%, 94.7%), respectively, and against COVID-19-related death were 85.7% (95% CI: 75.6%, 91.6%) and 96.2% (95% CI: 92.0%, 98.2%), respectively. Inactivated vaccine was 16.3% (95% CI: 15.4%, 17.2%) effective against infection, 88.6% (95% CI: 85.8%, 90.9%) effective against severe/critical COVIID-19, and 91.7% (95% CI: 86.95%, 94.7%) against COVID-19 death. Ad5-vectored vaccine was 13.2% (95% CI: 11.0%, 15.5%) effective against infection and 77.9% (95% CI: 15.6%, 94.2%) effective against severe/critical COVIID-19 (see Table 2).

**Table 2:** Vaccine effectiveness against documented SARS-CoV-2 infection, severe or critical illness, and death in matched case-control analysis by matching, age, gender, birthplace, date of illness onset, and residential district

Group	Documented SARS-CoV-2 infection, ***n*** = 612,597	Control 1, ***n*** = 612,597	VE (95% CI) (%)	Severe/critical illness, ***n*** = 1485	Control 2, ***n*** = 5940	VE (95% CI) (%)	Death, ***n*** = 568	Control 3, ***n*** = 2272	VE (95% CI) (%)
**Overall analysis**									
Unvaccinated	104,901 (17.1)	94,139 (15.4)	Reference	1312 (88.4)	3804 (64.0)	Reference	535 (94.2)	1639 (72.1)	Reference
With any vaccine	507,696 (82.9)	518,458 (84.6)	16.0 (15.1, 17.0)	173 (11.6)	2136 (36.0)	88.6 (85.8, 90.8)	33 (5.8)	633 (27.9)	91.6 (86.8, 94.6)
**Subgroup analysis**									
**By gender**									
Male^a^	289,105 (85.2)	295,602 (87.1)	18.9 (17.7, 20.2)	126 (15.2)	1505 (45.3)	88.0 (84.5, 90.7)	25 (8.3)	438 (36.4)	90.4 (84.1, 94.2)
Female^a^	218,591 (80.0)	222,856 (81.6)	13.0 (11.6, 14.3)	47 (7.2)	631 (24.1)	90.0 (84.6, 93.4)	8 (3.0)	195 (18.3)	94.6 (84.9, 98.0)
**By age**									
3–17 years^a^	25,875 (72.8)	26,861 (75.6)	20.1 (16.7, 23.4)	0 (0.0)	4 (100.0)	90.6^b^ (54.0, 98.1)	–	–	–
18–39 years^a^	198,590 (91.6)	198,175 (91.4)	− 2.6 (− 4.8, − 0.4)	6 (46.1)	44 (84.6)		1 (50.0)	8 (100.0)	93.4^c^ (76.8, 98.1)
40–59 years^a^	209,271 (91.6)	210,530 (92.2)	7.5 (5.5, 9.5)	47 (49.5)	344 (90.5)	94.8 (88.4, 97.7)	8 (36.4)	78 (88.6)	
60–79 years^a^	71,698 (63.7)	79,695 (70.8)	29.8 (28.5, 31.1)	90 (17.8)	1288 (63.6)	89.6 (86.3, 92.0)	18 (12.2)	322 (54.8)	90.0 (82.3, 94.3)
80+ years^a^	2262 (11.8)	3206 (16.6)	36.7 (32.6, 40.5)	30 (3.4)	456 (13.1)	79.8 (69.8, 86.4)	6 (1.51)	225 (14.2)	93.5 (83.8, 97.4)
**By residential district**									
Urban^a^	174,836 (77.5)	181,748 (80.5)	22.1 (20.8, 23.4)	83 (10.2)	1121 (34.3)	88.1 (84.1, 91.1)	12 (3.8)	318 (25.3)	93.6 (86.8, 96.9)
Rural^a^	332,860 (86)	336,710 (87.0)	10.7 (9.4, 12.1)	90 (13.5)	1015 (37.9)	89.1 (84.9, 92.1)	21 (8.3)	315 (31.0)	89.6 (81.5, 94.2)
**By vaccination course**									
Unvaccinated	104,901 (17.1)	94,139 (15.4)	Reference	1312 (88.4)	3804 (64.0)	Reference	535 (94.2)	1639 (72.1)	Reference
Partial vaccination	14,809 (2.4)	13,376 (2.2)	4.4 (1.9, 6.8)	10 (0.7)	67 (1.1)	74.7 (49.1, 87.5)	2 (0.4)	26 (1.1)	80.4 (16.4, 95.4)
Full vaccination	219,178 (35.8)	220,764 (36.0)	14.7 (13.7, 15.7)	96 (6.5)	866 (14.6)	83.8 (79.0, 87.5)	23 (4.0)	257 (11.3)	85.7 (75.6, 91.6)
Booster vaccination	273,709 (44.7)	284,318 (46.4)	18.0 (17.0, 18.9)	67 (4.5)	1203 (20.3)	92.8 (90.2, 94.7)	8 (1.4)	350 (15.4)	96.2 (92.0, 98.2)
**By vaccine type**				10 (0.7)	67 (1.1)	74.7 (49.1, 87.5)	2 (0.4)	26 (1.1)	80.4 (16.4, 95.4)
Unvaccinated	104,901 (17.1)	94,139 (15.4)	Reference	1312 (88.4)	3804 (64.0)	Reference	535 (94.2)	1639 (72.1)	Reference
Inactivated vaccine	485,190 (79.2)	498,662 (81.4)	16.3 (15.4, 17.2)	170 (11.4)	2114 (35.6)	88.6 (85.8, 90.9)	32 (5.6)	622 (27.4)	91.7 (86.9, 94.7)
Adenovirus vector vaccine	14,022 (2.3)	13,817 (2.3)	13.2 (10.9, 15.5)	3 (0.2)	16 (0.3)	77.9 (15.6, 94.2)	1 (0.2)	4 (0.2)	NA^d^
Recombined protein vaccine	8484 (1.4)	5979 (1.0)	NA^d^	0 (0.0)	6 (0.1)	NA^d^	0 (0.0)	7 (0.3)	NA^d^

VEs were from univariate conditional logistic regression and were unadjusted for baseline characteristics for the exact matching process. The effectiveness of at least one dose of COVID-19 vaccines was estimated in the whole population and in subgroups defined by strata of age, gender, and residential district. The effectiveness of each vaccination course was estimated in the whole population

^a^The proportion of vaccinated individuals was calculated as the number of vaccinated people divided by the total number of people in the corresponding category

^b^Vaccine effectiveness for the combined age group 3–39 years

^c^Vaccine effectiveness for the combined age group 18–59 years

^d^Vaccine effectiveness in this cell had an infinite confidence interval

**Fig. 3:**
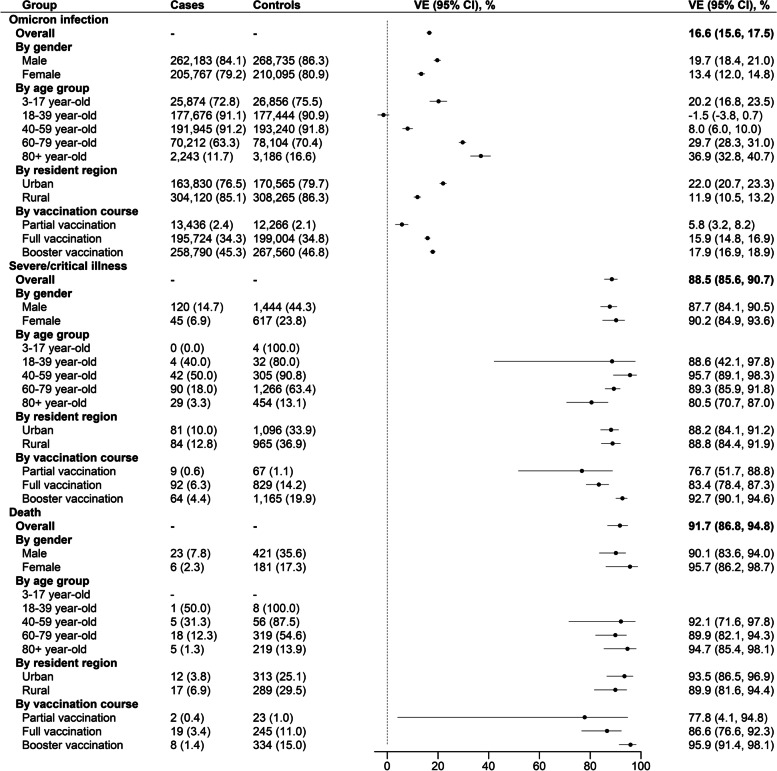
Vaccine effectiveness of inactivated vaccines against documented SARS-CoV-2 infection, severe or critical illness, and COVID-19 death in the matched case-control study. VEs were from univariate conditional logistic regression and were unadjusted for baseline characteristics for the exact matching process. The effectiveness of at least one dose of inactivated vaccines was estimated in the whole population and in subgroups defined by strata of age, gender, and resident region. The effectiveness of each vaccination course was estimated in the whole population

We used subset 1 and subset 2 to estimate the vaccine-specific VEs. Figure 3 shows the VEs of inactivated vaccine against SARS-CoV-2 infection, severe/critical illness, and COVID-19-related death; respective overall VEs were 16.6% (95% CI: 15.6%, 17.5%), 88.5% (95% CI:85.6%, 90.7%), and 91.7 (95% CI: 86.8%, 94.8%). Booster vaccination with inactivated vaccine enhanced the protection for both severe outcome (VE: 92.7%, 95% CI: 90.1%, 94.6%) and death (VE: 95.9%, 95% CI: 91.4%, 98.1%).

**Fig. 4:**
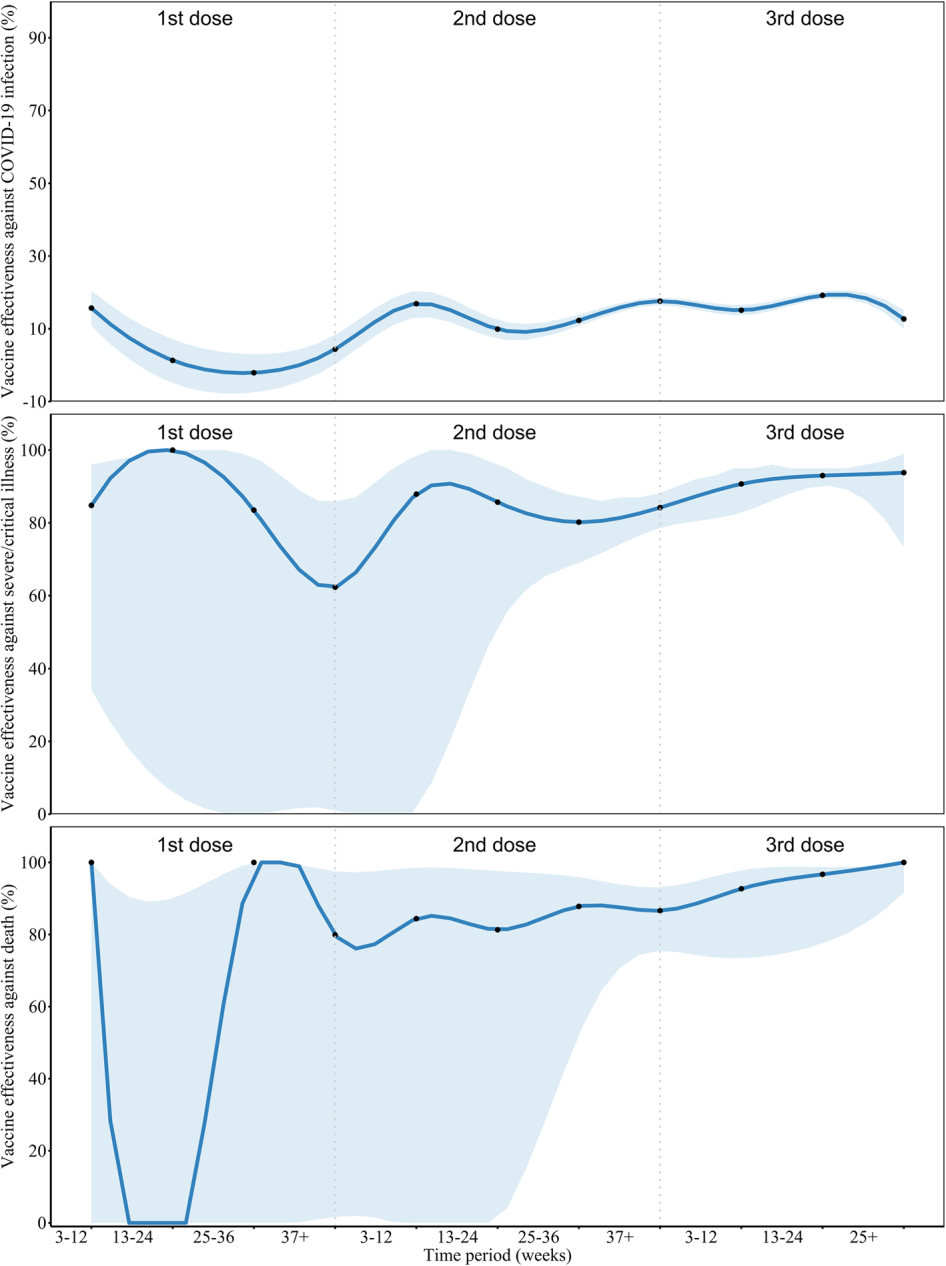
VE (%) of inactivated vaccine against documented SARS-CoV-2 infection, severe or critical illness, and death by doses and time interval. Data are presented with VEs (black points) and 95% confidence intervals (color zones). To visualize the trend of vaccine effectiveness (dark blue lines), we smoothed the VE results by performing three spline interpolations of the calculated data points for the corresponding time period. Small sample sizes of corresponding cells lead to wide confidence intervals. Eight individuals had severe/critical illness and two died within first-dose intervals

Considering the time between the last dose and SARS-Co2 exposure, Fig. 4 shows that the VE of inactivated vaccines against SARS-CoV-2 infection was 15.7% (95% CI: 10.8%, 20.4%) 3–12 weeks after the first dose. Two-dose inactivated vaccine offered 16.9% (95% CI: 13.2%, 20.4%) protection for infection after 12 weeks, and three-dose offered 19.2% (95% CI: 18.2%, 20.3%) after 24 weeks, then both subsequently becoming lower. Full-series and boosted VEs for severe/critical illness and COVID-19 death remained > 80%.

For SARS-CoV-2 infection, estimated VEs of Ad5-vectored vaccine were 39.3% (95% CI: 17.9%, 68.8%) at 3–12 weeks after the first dose and 16.0% (95% CI: 1.5%, 28.4%) at 13–24 weeks after the second dose. For severe/critical illness, just one case and one control received vaccine, and for COVID-19-related death, one case and zero control received vaccine. Thus, VE estimation was not available (Additional file 1: Table S2).

### Sensitivity analysis

Additional file 1: Tables S3 and S4 show the sensitivity analyses of VEs which were consistent with the results displayed in Table 2 and Fig. 3.

## Discussion

From 2 December 2021 to 13 May 2022, during an Omicron BA 2.2-predominant wave of COVID-19 in Shanghai, China, we used detailed, individual-level, record-documented COVID-19 vaccination status; universal, population-wide individual-level SARS-CoV-2 RT-PCR testing results; and individual-level clinical evaluations to estimate the real-world effectiveness of inactivated and Ad5-vectored vaccines in a largely infection-naïve population. Our matched case-control study showed that the inactivated vaccines were highly effective against severe or critical COVID-19 and COVID-19-related death, findings that underscore the potential of the vaccines to save lives and substantially reduce demands on the healthcare system. Inactivated vaccine was 16.3% effective against SARS-CoV-2infection, 88.6% effective against severe/critical COVID-19, and 91.7% against COVID-19 death. Ad5-vectored vaccine was 13.2% effective against infection and 77.9% effective against severe/critical COVID-19. Booster-dose protection against severe/critical COVID-19 was 92.7% and against COVID-19 death was 95.9%. Protection from infection waned after 12 weeks, but primary-series and booster-dose protection was sustained above 80% for at least 36 weeks from the last dose.

We found that VE against SARS-CoV-2 infection was lower than the efficacy against ancestral strain SARS-CoV-2 symptomatic infection observed in a phase 3 clinical trial (72.8% and 78.1%) and a real-world, pre-Omicron VE study in Chile (83.5%) [16, 17]. However, our VE estimates for the two predominant inactivated (Sinovac and Sinopharm) vaccines were very high in age group analyses, notably among persons 80 years of age or older and independent of time since vaccination. The lower VE against SARS-CoV-2 infection in younger adults may be partly due to a higher force of infection in working-age adults or differences in willingness to be vaccinated (although vaccination rates were very high in all ages under 60 years) [18].

Booster vaccination was 93% and 96% effective against severe illness and death, respectively, somewhat higher than full primary-series vaccination VEs. We found that inactivated VE against severe illness and COVID-19-associated death is higher than respective VEs observed in Brazil (77.6% and 83.9%) [19, 20] and Chile (90.3% and 86.3%) [17], in which the circulating SARS-CoV-2 strains predated Omicron emergence. Clinical trials have shown inactivated vaccine efficacies to be lower than that of mRNA vaccines, but there are fewer real-world data on two- and three-dose inactivated VE against Omicron infection or hospitalization [16, 21]. The Hong Kong ecological study found an initial VE from two doses of inactivated COVID-19 vaccine against Omicron infection to be 17.9% with rapid waning—similar to our results [14]. Also, similar to our findings, the Hong Kong scientists showed inactivated VE against severe illness and death to be very high—74% for primary vaccination and 98% for boosted vaccination. Our study extended the time frame for the duration of inactivated VE, showing sustained protection against severe COVID-19 and COVID-19 death through 36 weeks.

Omicron BA. 2 COVID-19 disease is less severe than Delta and BA. 1-associated COVID-19 disease [22], resulting in a lower risk of hospitalization and severe illness. Indeed, over 85% of the SARS-CoV-2 infections reported from Shanghai by China’s National Health Commission were asymptomatic (http://www.nhc.gov.cn/xcs/xxgzbd/gzbd_index.shtml). Milder infections may reflect increased replication of Omicron in the upper versus lower respiratory tract—a phenomenon that could contribute to more efficient transmission, leading to a higher absolute number of hospitalizations. Omicron infection enhances preexisting immunity elicited by vaccines but, on its own, may not confer broad protection against non-Omicron variants in unvaccinated individuals [23]. A study demonstrated that inactivated vaccines elicited SARS-CoV-2-specific memory T/B cell immunity, which is important for robust recall of protective responses against viral replication and therefore induces longer-term protection against severe illness and deaths [24, 25]. However, Omicron’s immune evasion, including evasion of mucosal immunity, allows Omicron to escape existing antibodies and increase the risk of reinfection, leading to a surge in infections [26, 27]. Evaluations of vaccines that have the potential to block infection/transmission (e.g., the inhaled Ad5-vectored vaccine by CanSino that may provide mucosal immunity) are especially important [28, 29].

We estimated the VEs of the Ad5-vectored vaccine to be 39.3% at 3–12 weeks after the first dose but 16.0% at 13–24 weeks after the second dose. A study in Brazil reported that whereas waning of VE against symptomatic COVID-19 was observed ≥ 90 days after homologous and heterologous boosters, waning against severe COVID-19 was only observed after a homologous booster, suggesting a need for adjustment of vaccination strategies—e.g., heterologous boosters for those who have received 2 doses of inactivated vaccines as their primary series [20]. None of the current COVID-19 vaccines blocks infection/transmission; our results also show that the vaccines we evaluated cannot make a permanent barrier to contain the SARS-CoV-2 transmission in the community, despite high vaccine coverage.

Our study has strengths. The setting was a COVID-19 outbreak in the large and diverse socioeconomic population of Shanghai in which 2.7% of the population was infected during the Omicron BA. 2 variant of SARS-CoV-2-dominant period before the outbreak was stopped by public health and social interventions. In addition, because COVID-19 is being managed as a level 1 infectious disease, all infected individuals are compulsively reported to the disease prevention and control system daily. Infections are always confirmed with additional RT-PCR testing, making diagnostic misclassification highly unlikely. The cases in our study were from a community of 25 million people, repeatedly screened for infection using RT-PCR. We used individual-level exact matching to control for potential confounding factors of age, sex, residential district, birthplace, and illness onset time. Finally, our study measured VE levels in an infection-naïve population, allowing assessment of nearly pure vaccine-induced, real-world VE, avoiding distortion from hybrid immunity.

Limitations of our study include possible misclassification of disease status, which was judged by the COVID-19-designated hospitals. The definitions of severe/critical COVID-19 are objective, and the misclassification of severe illness is unlikely because disease severity for all SARS-CoV-2 infections is made in accordance with the *Diagnosis and Treatment Protocol for COVID-19*. A second limitation is the lack of information on comorbidities, precluding analyses by comorbidity. We attempted to reduce potential bias by matching sociodemographic characteristics. In addition, the younger-aged population is at the highest risk of infection and may also have a greater willingness or opportunity to get vaccinated, possibly leading to an underestimated VE among working-age adults. Third, the study was conducted during a strict lockdown and with parallel non-pharmaceutical interventions—especially after April 4—which may have biased the estimation of VE (e.g., more likely to have a null hypothesis) by decreasing the force of infection (attack rate among susceptibles). Meanwhile, the suspended vaccination campaign especially the booster vaccination may also bias towards reduced VE. However, universal NAAT screenings and exact matching that included district helped ensure similar exposure risk of cases and controls, and our sensitivity analysis showed no meaningful differences in the results. Finally, the number of Ad5-vectored vaccine doses used in Shanghai was too small for subgroup analyses and for estimating VE against death.

## Conclusions

Our study showed high and durable two- and three-dose inactivated VE against severe/critical COVID-19 and death during Omicron BA. 2 variant dominantly prevalent period infection across all age groups, but with lower effectiveness against Omicron infection. Everyone eligible should be vaccinated; individuals eligible for booster doses should receive timely boosters; improving coverage among the elderly is especially important. The vaccines being used in China are highly effective where it matters most—preventing severe illness and death—and are most effective when boosted.

## Supplementary Information

**Additional file 1: Table S1.** The definitions of the variables. **Table S2.** Vaccine effectiveness of Ad5-vectored vaccine against documented SARS-CoV-2 infection, severe or critical illness and death in matched case-control analysis. **Table S3.** Sensitivity analysis for vaccine effectiveness of any vaccine in matched case-control assuming exposure window 7 days prior to onset date. **Table S4.** Sensitivity analysis for vaccine effectiveness of inactivated vaccines in matched case-control analysis assuming exposure window 7 days prior to onset date. **Figure S1.** Subset 1 for inactivated vaccines estimates of effectiveness against documented SARS-CoV-2 infection, severe/critical illness and Covid-19 related death. **Figure S2.** Subset 2 for Ad5-nCoV vaccine estimates of effectiveness against documented SARS-CoV-2 infection, severe/critical illness, and Covid-19 related death. **Figure S3.** The process of vaccination history retrieval.

## Data Availability

The dataset used for this study is available through a request to Zhuoying Huang (e-mail: huangzhuoying@scdc.sh.cn), Dr. Xiaodong Sun (e-mail: sunxiaodong@scdc.sh.cn), and Dr. Weibing Wang (e-mail: wwb@fudan.edu.cn).

## Abbreviations

BPM: Breaths per minute
CI: Confidence intervals
COVID-19: Coronavirus disease 2019
IQR: Interquartile ranges
NAAT: Nucleic acid amplification testing
NNDRS: National Notifiable Diseases Registry System
OR: Odds ratio
RR: Respiration rate
RT-PCR: Real-time polymerase chain reaction
SARS-CoV-2: Severe acute respiratory syndrome coronavirus 2
VE: Vaccine effectiveness

## References

[1] World Health Organization (2022). Statement on Omicron sublineage BA. 2.

[2] Zhang X, Zhang W, Chen S. Shanghai’s life-saving efforts against the current omicron wave of the COVID-19 pandemic. Lancet (London, England). 2022;399(10340):S0140-6736(22)00838-8. 10.1016/S0140-6736(22)00838-8 PMC9075855 35533708

[3] Viana R, Moyo S, Amoako DG (2022). Rapid epidemic expansion of the SARS-CoV-2 Omicron variant in southern Africa. Nature.

[4] Uraki R, Kiso M, Iida S (2022). Characterization and antiviral susceptibility of SARS-CoV-2 Omicron/BA. 2. Nature.

[5] Yu J, Collier AY, Rowe M (2022). Neutralization of the SARS-CoV-2 Omicron BA. 1 and BA. 2 variants. N Engl J Med.

[6] Dagan N, Barda N, Kepten E (2021). BNT162b2 mRNA COVID-19 vaccine in a nationwide mass vaccination setting. N Engl J Med.

[7] Haas EJ, Angulo FJ, McLaughlin JM (2021). Impact and effectiveness of mRNA BNT162b2 vaccine against SARS-CoV-2 infections and COVID-19 cases, hospitalisations, and deaths following a nationwide vaccination campaign in Israel: an observational study using national surveillance data. Lancet.

[8] Pawlowski C, Lenehan P, Puranik A (2021). FDA-authorized mRNA COVID-19 vaccines are effective per real-world evidence synthesized across a multi-state health system. Med (N Y).

[9] Andrews N, Stowe J, Kirsebom F (2022). COVID-19 vaccine effectiveness against the Omicron (B.1.1.529) variant. N Engl J Med.

[10] Chemaitelly H, Yassine HM, Benslimane FM (2021). mRNA-1273 COVID-19 vaccine effectiveness against the B.1.1.7 and B.1.351 variants and severe COVID-19 disease in Qatar. Nat Med.

[11] Wright BJ, Tideman S, Diaz GA, French T, Parsons GT, Robicsek A (2022). Comparative vaccine effectiveness against severe COVID-19 over time in US hospital administrative data: a case-control study. Lancet Respir Med.

[12] Cao Y, Wang J, Jian F (2022). Omicron escapes the majority of existing SARS-CoV-2 neutralizing antibodies. Nature.

[13] Premikha M, Chiew CJ, Wei WE (2022). Comparative effectiveness of mRNA and inactivated whole virus vaccines against COVID-19 infection and severe disease in Singapore. Clin Infect Dis.

[14] McMenamin ME, Nealon J, Lin Y, et al. Vaccine effectiveness of one, two, and three doses of BNT162b2 and CoronaVac against COVID-19 in Hong Kong: a population-based observational study. Lancet Infect Dis. 2022;22(10):1435-43. 10.1016/S1473-3099(22)00345-0 PMC9286709 35850128

[15] National Health Commission & State Administration of Traditional Chinese Medicine (2022). Diagnosis and treatment protocol for COVID-19 (trial version 9).

[16] Al Kaabi N, Zhang Y, Xia S (2021). Effect of 2 inactivated SARS-CoV-2 vaccines on symptomatic COVID-19 infection in adults: a randomized clinical trial. JAMA.

[17] Jara A, Undurraga EA, González C (2021). Effectiveness of an inactivated SARS-CoV-2 vaccine in Chile. N Engl J Med.

[18] Nasreen S, Chung H, He S (2022). Effectiveness of COVID-19 vaccines against symptomatic SARS-CoV-2 infection and severe outcomes with variants of concern in Ontario. Nat Microbiol.

[19] Ranzani OT, Hitchings MDT, Dorion M (2021). Effectiveness of the CoronaVac vaccine in older adults during a gamma variant associated epidemic of COVID-19 in Brazil: test negative case-control study. BMJ.

[20] Ranzani OT, Hitchings MDT, de Melo RL, et al. Effectiveness of an inactivated Covid-19 vaccine with homologous and heterologous boosters against Omicron in Brazil. Nat Commun. 2022;13(1):5536. 10.1038/s41467-022-33169-0 PMC9537178 36202800

[21] Wilder-Smith A, Mulholland K (2021). Effectiveness of an inactivated SARS-CoV-2 vaccine. N Engl J Med.

[22] Wolter N, Jassat W, Walaza S (2022). Early assessment of the clinical severity of the SARS-CoV-2 omicron variant in South Africa: a data linkage study. Lancet.

[23] Suryawanshi RK, Chen IP, Ma T, et al. Limited Cross-Variant Immunity after Infection with the SARS-CoV-2 Omicron Variant Without Vaccination. Preprint. medRxiv. 2022;2022.01.13.22269243. Published 2022 Feb 9. 10.1101/2022.01.13.22269243.

[24] Liu Y, Zeng Q, Deng C (2022). Robust induction of B cell and T cell responses by a third dose of inactivated SARS-CoV-2 vaccine. Cell Discov.

[25] Ahmed SF, Quadeer AA, McKay MR (2022). SARS-CoV-2 T cell responses elicited by COVID-19 vaccines or infection are expected to remain robust against Omicron. Viruses.

[26] Ao D, Lan T, He X (2022). SARS-CoV-2 Omicron variant: immune escape and vaccine development. MedComm (2020).

[27] Lapuente D, Fuchs J, Willar J (2021). Protective mucosal immunity against SARS-CoV-2 after heterologous systemic prime-mucosal boost immunization. Nat Commun.

[28] Schubert M, Bertoglio F, Steinke S (2022). Human serum from SARS-CoV-2-vaccinated and COVID-19 patients shows reduced binding to the RBD of SARS-CoV-2 Omicron variant. BMC Med.

[29] Bartsch YC, Tong X, Kang J (2022). Omicron variant Spike-specific antibody binding and Fc activity are preserved in recipients of mRNA or inactivated COVID-19 vaccines. Sci Transl Med.

