# Supplementary material for: Effectiveness of inactivated and Ad5-nCoV COVID-19 vaccines against SARS-CoV-2 Omicron BA. 2 variant infection, severe illness, and death
Source: BMC Med. 2022 Oct 20;20:400. doi: 10.1186/s12916-022-02606-8 (PMC9583051; doi:10.1186/s12916-022-02606-8)
Supplement: Supplementary file 1 — Additional file 1: Table S1. The definitions of the variables. Table S2. Vaccine effectiveness of Ad5-vectored vaccine against documented SARS-CoV-2 infection, severe or critical illness and death in matched case-control analysis. Table S3. Sensitivity analysis for vaccine effectiveness of any vaccine in matched case-control assuming exposure window 7 days prior to onset date. Table S4. Sensitivity analysis for vaccine effectiveness of inactivated vaccines in matched case-control analysis assuming exposure window 7 days prior to onset date. Figure S1. Subset 1 for inactivated vaccines estimates of effectiveness against documented SARS-CoV-2 infection, severe/critical illness and Covid-19 related death. Figure S2. Subset 2 for Ad5-nCoV vaccine estimates of effectiveness against documented SARS-CoV-2 infection, severe/critical illness, and Covid-19 related death. Figure S3. The process of vaccination history retrieval. [file 12916_2022_2606_MOESM1_ESM.docx]

Additional Files

| Table S1. The definitions of the variables | Page 2 |
| --- | --- |
| Table S2. Vaccine effectiveness of Ad5-vectored vaccine against documented SARS-CoV-2 infection, severe or critical illness and death in matched case-control analysis | Page 3 |
| Table S3. Sensitivity analysis for vaccine effectiveness of any vaccine in matched case-control assuming exposure window 7 days prior to onset date | Page 4 |
| Table S4. Sensitivity analysis for vaccine effectiveness of inactivated vaccines in matched case-control analysis assuming exposure window 7 days prior to onset date | Page 5 |
| Figure S1. Subset 1 for inactivated vaccines estimates of effectiveness against documented SARS-CoV-2 infection, severe/critical illness and Covid-19 related death | Page 7 |
| Figure S2. Subset 2 for Ad5-nCoV vaccine estimates of effectiveness against documented SARS-CoV-2 infection, severe/critical illness, and Covid-19 related death | Page 8 |
| Figure S3. The process of vaccination history retrieval | Page 9 |

Table S1 The definitions of the variables

| **Variable** | **Values** |
| --- | --- |
| Age | Integer |
| Age group | 3-17 years/18-39 years/40-59 years/60-79 years/≥80 years |
| Gender | Male/Female |
| Residential district | Urban/Rural |
| Vaccination | Unvaccinated (reference level)/vaccinated |
| Vaccination course | Unvaccinated (reference level)/Partial vaccination/Full vaccination/Booster vaccination |
| Vaccine type | Unvaccinated (reference level)/  Inactivated vaccine/Adenovirus vector vaccine/Recombined protein vaccine |
| Vaccination intervals (weeks) | Unvaccinated (reference level)/  Dose1: 0~2/Dose1: 3~12/Dose1: 13~24/Dose1: 25~36/Dose1: 37+/  Dose2: 0~2/Dose2: 3~12/Dose 2: 13~24/Dose 2: 25~36/Dose 2: 37+/  Dose 3: 0~2/Dose 3: 3~12/Dose 3: 13~24/Dose 3: 25+ |

Table S2. Vaccine effectiveness of Ad5-vectored vaccine against documented SARS-CoV-2 infection in matched case-control analysis in subset 2

| **Group** | **Documented SARS-CoV-2 infection** | | |
| --- | --- | --- | --- |
|  | **Infected cases**  **(n = 40032)** | **Control**  **(n = 40032)** | **VE (95% CI)**  **(%)** |
| **Unvaccinated** | 38439 (96.0) | 38434 (96.0) | Reference |
| **Vaccinated** | 1593 (4.0) | 1598 (4.0) | 0.4 (-8.1, 8.3) |
| **By gender** |  |  |  |
| Male ^a^ | 1069 (6.1) | 1070 (6.1) | 0.1 (-10.8, 10.0) |
| Female ^a^ | 524 (2.3) | 528 (2.3) | 0.9 (-13.3, 13.4) |
| **By age** |  |  |  |
| 3-17 years ^a^ | 1 (0.0) | 0 (0.0) | NA ^a^ |
| 18-39 years ^a^ | 975 (27.2) | 905 (25.2) | NA ^a^ |
| 40-59 years ^a^ | 502 (15.5) | 493 (15.2) | -2.5 (-18.5, 11.4) |
| 60-79 years ^a^ | 105 (0.7) | 192 (1.3) | 46.3 (31.6, 57.8) |
| 80+ years ^a^ | 10 (0.1) | 8 (0.1) | -25.0 (-216.7, 50.7) |
| **By residential district** |  |  |  |
| Urban ^a^ | 450 (2.2) | 577 (2.8) | 27.4 (16.4, 36.9) |
| Rural ^a^ | 1143 (5.8) | 1021 (5.2) | -18.0 (-30.7, -6.5) |
| **By vaccination course** |  |  |  |
| Partial vaccination | - | - | - |
| Full vaccination | 1120 (2.8) | 1054 (2.6) | -5.8 (-16.2, 3.7) |
| Booster vaccination | 473 (1.2) | 544 (1.4) | 13.2 (1.0, 23.9) |
| **By vaccination intervals (weeks)** |  |  |  |
| Dose1: 0~2 | - | - | - |
| Dose1: 3~12 | 14 (0.0) | 23 (0.1) | 39.3 (-17.9, 68.8) |
| Dose1: 13~24 | 45 (0.1) | 56 (0.1) | 19.5 (-19.4, 45.7) |
| Dose1: 25+ | 1061 (2.7) | 975 (2.4) | -8.5 (-19.5, 1.5) |
| Dose2: 0~2 | 3 (0.0) | 4 (0.0) | 23.2 (-243.1, 82.8) |
| Dose2: 3~12 | 148 (0.4) | 171 (0.4) | 13.5 (-8.3, 30.9) |
| Dose 2: 13~24 | 308 (0.8) | 365 (0.9) | 16.0 (1.5, 28.4) |
| Dose 2: 25+ | 14 (0.0) | 4 (0.0) | NA ^a^ |

a: Vaccine effectiveness in this cell had an infinite confidence interval

Table S3. Sensitivity analysis for vaccine effectiveness of any vaccine in matched case-control assuming exposure window 7 days prior to onset date

| **Group** | **Documented SARS-CoV-2 infection** | | |  | **Severe/critical illness** | | |  | **Death** | | |
| --- | --- | --- | --- | --- | --- | --- | --- | --- | --- | --- | --- |
|  | **Infected cases**  **(n = 612597)** | **Control**  **(n = 612597)** | **VE (95% CI)**  **(%)** |  | **Severe or critical cases**  **(n = 1485** | **Control**  **(n = 5940)** | **VE (95% CI)**  **(%)** |  | **Died cases**  **(n = 568)** | **Control**  **(n = 2272)** | **VE (95% CI)**  **(%)** |
| **Unvaccinated** | 104907 (17.1) | 94166 (15.4) | Reference |  | 1312 (88.4) | 3804 (64.0) | Reference |  | 535 (94.2) | 1639 (72.1) | Reference |
| **With any vaccine** | 507690 (82.9) | 518431 (84.6) | 16.0 (15.0, 16.9) |  | 173 (11.6) | 2136 (36.0) | 88.6 (85.8, 90.8) |  | 33 (5.8) | 633 (27.9) | 91.6 (86.8, 94.6) |
| **By gender** |  |  |  |  |  |  |  |  |  |  |  |
| Male ^a^ | 289102 (85.2) | 295591 (87.1) | 18.9 (17.6, 20.2) |  | 126 (15.2) | 1505 (45.3) | 88.0 (84.5, 90.7) |  | 25 (8.3) | 438 (36.4) | 90.4 (84.1, 94.2) |
| Female ^a^ | 218588 (80.0) | 222840 (81.6) | 12.9 (11.5, 14.3) |  | 47 (7.2) | 631 (24.1) | 90.0 (84.6, 93.4) |  | 8 (3.0) | 195 (18.3) | 94.6 (84.9, 98.0) |
| **By age** |  |  |  |  |  |  |  |  |  |  |  |
| 3-17 years ^a^ | 25875 (72.8) | 26850 (75.5) | 20 (16.6, 23.3) |  | 0 (0.0) | 4 (100.0) | 90.6^b^ (54.0, 98.1) |  | - | - | - |
| 18-39 years ^a^ | 198587 (91.6) | 198164 (91.4) | -2.6 (-4.9, -0.4) |  | 6 (46.1) | 44 (84.6) |  |  | 1 (50.0) | 8 (100.0) | 93.4^c^ (76.8, 98.1) |
| 40-59 years ^a^ | 209270 (91.6) | 210521 (92.2) | 7.5 (5.4, 9.5) |  | 47 (49.5) | 344 (90.5) | 94.8 (88.4, 97.7) |  | 8 (36.4) | 78 (88.6) |  |
| 60-79 years ^a^ | 71696 (63.7) | 79690 (70.8) | 29.8 (28.5, 31.1) |  | 90 (17.8) | 1288 (63.6) | 89.6 (86.3, 92.0) |  | 18 (12.2) | 322 (54.8) | 90.0 (82.3, 94.3) |
| 80+ years ^a^ | 2262 (11.7) | 3206 (16.7) | 36.7 (32.6, 40.5) |  | 30 (3.4) | 456 (13.1) | 79.8 (69.8, 86.4) |  | 6 (1.51) | 225 (14.2) | 93.5 (83.8, 97.4) |
| **By** **residential district** |  |  |  |  |  |  |  |  |  |  |  |
| Urban ^a^ | 174834 (77.5) | 181739 (80.5) | 22.1 (20.8, 23.4) |  | 83 (10.2) | 1121 (34.3) | 88.1 (84.1, 91.1) |  | 12 (3.8) | 318 (25.3) | 93.6 (86.8, 96.9) |
| Rural ^a^ | 332856 (86.0) | 336692 (87.0) | 10.7 (9.3, 12.0) |  | 90 (13.5) | 1015 (37.9) | 89.1 (84.9, 92.1) |  | 21 (8.3) | 315 (31.0) | 89.6 (81.5, 94.2) |
| **By vaccination course** |  |  |  |  |  |  |  |  |  |  |  |
| Partial vaccination | 14803 (2.4) | 13354 (2.2) | 4.2 (1.7, 6.7) |  | 10 (0.7) | 67 (1.1) | 74.7 (49.1, 87.5) |  | 2 (0.4) | 26 (1.1) | 80.4 (16.4, 95.4) |
| Full vaccination | 219178 (35.8) | 220859 (36.1) | 14.7 (13.7, 15.8) |  | 96 (6.5) | 866 (14.6) | 83.8 (79.0, 87.5) |  | 23 (4.0) | 257 (11.3) | 85.7 (75.6, 91.6) |
| Booster vaccination | 273709 (44.7) | 284218 (46.4) | 17.9 (16.9, 18.9) |  | 67 (4.5) | 1203 (20.3) | 92.8 (90.2, 94.7) |  | 8 (1.4) | 350 (15.4) | 96.2 (92.0, 98.2) |
| **By Vaccine type** |  |  |  |  | 10 (0.7) | 67 (1.1) | 74.7 (49.1, 87.5) |  | 2 (0.4) | 26 (1.1) | 80.4 (16.4, 95.4) |
| Inactivated vaccine | 485185 (79.2) | 498639 (81.4) | 16.3 (15.3, 17.2) |  | 170 (11.4) | 2114 (35.6) | 88.6 (85.8, 90.9) |  | 32 (5.6) | 622 (27.4) | 91.7 (86.9, 94.7) |
| Adenovirus vector vaccine | 14021 (2.3) | 13814 (2.3) | 13.2 (10.9, 15.4) |  | 3 (0.2) | 16 (0.3) | 77.9 (15.6, 94.2) |  | 1 (0.2) | 4 (0.2) | NA^d^ |
| Recombined protein vaccine | 8484 (1.4) | 5978 (1.0) | NA^d^ |  | 0 (0.0) | 6 (0.1) | NA^d^ |  | 0 (0.0) | 7 (0.3) | NA^d^ |

VEs were from univariate conditional logistic regression and were unadjusted for baseline characteristics for exact matching process. Effectiveness of at least one dose Covid-19 vaccines was estimated in the whole population and in subgroups defined by strata of age, gender, and residential district. Effectiveness of each vaccination course was estimated in the whole population.

a: The proportion of vaccinated individuals were calculated as the number of vaccinated people divided by the total number of people in corresponding category.

b: Vaccine effectiveness for the combined age group 3-39 years.

c: Vaccine effectiveness for the combined age group 18-59 years.

d: Vaccine effectiveness in this cell had an infinite confidence interval

Table S4. Sensitivity analysis for vaccine effectiveness of inactivated vaccines in matched case-control analysis assuming exposure window 7 days prior to onset date

| **Group** | **Documented SARS-CoV-2 infection** | | |  | **Severe/critical illness** | | | |  | | **Death** | | | |
| --- | --- | --- | --- | --- | --- | --- | --- | --- | --- | --- | --- | --- | --- | --- |
|  | **Infected cases**  **(n = 571311)** | **Control**  **(n = 571311)** | **VE (95% CI)**  **(%)** |  | **Severe or critical cases**  **(n = 1462)** | **Control**  **(n = 5848)** | **VE (95% CI)**  **(%)** |  | | **Died cases**  **(n = 557)** | | **Control**  **(n = 2228)** | **VE (95% CI)**  **(%)** |  |
| **Unvaccinated** | 103362 (18.1) | 92503 (16.2) | Reference |  | 1297 (88.7) | 3787 (64.8) | Reference |  | | 528 (94.8) | | 1626 (73.0) | Reference |  |
| **Vaccinated** | 467949 (81.9) | 478808 (83.8) | 16.5 (15.6, 17.5) |  | 165 (11.3) | 2061 (35.2) | 88.5 (85.6, 90.7) |  | | 29 (5.2) | | 602 (27.0) | 91.7 (86.8, 94.8) |  |
| **By gender** |  |  |  |  |  |  |  |  | |  | |  |  |  |
| Male ^a^ | 262184 (84.1) | 268728 (86.2) | 19.6 (18.3, 20.9) |  | 120 (14.7) | 1444 (44.3) | 87.7 (84.1, 90.5) |  | | 23 (7.8) | | 421 (35.6) | 90.1 (83.6, 94.0) |  |
| Female ^a^ | 205765 (79.2) | 210080 (80.9) | 13.4 (12, 14.7) |  | 45 (6.9) | 617 (23.8) | 90.2 (84.9, 93.6) |  | | 6 (2.3) | | 181 (17.3) | 95.7 (86.2, 98.7) |  |
| **By age** |  |  |  |  |  |  |  |  | |  | |  |  |  |
| 3-17 years ^a^ | 25874 (72.8) | 26850 (75.5) | 20.0 (16.6, 23.3) |  | 0 (0.0) | 4 (100.0) | 88.6 ^b^ (42.1, 97.8) |  | | - | | - |  |  |
| 18-39 years ^a^ | 177676 (91.1) | 177436 (90.9) | -1.6 (-3.8, 0.7) |  | 4 (40.0) | 32 (80.0) |  |  | | 1 (50.0) | | 8 (100.0) | 92.1 ^c^ (71.6, 97.8) |  |
| 40-59 years ^a^ | 191946 (91.2) | 193236 (91.8) | 8.0 (5.9, 10.0) |  | 42 (50.0) | 305 (90.8) | 95.7 (89.1, 98.3) |  | | 5 (31.3) | | 56 (87.5) |  |  |
| 60-79 years ^a^ | 70210 (63.3) | 78100 (70.4) | 29.7 (28.3, 31) |  | 90 (18.0) | 1266 (63.4) | 89.3 (85.9, 91.8) |  | | 18 (12.3) | | 319 (54.6) | 89.9 (82.1, 94.3) |  |
| 80+ years ^a^ | 2243 (11.7) | 3186 (16.6) | 36.9 (32.8, 40.7) |  | 29 (3.3) | 454 (13.1) | 80.5 (70.7, 87.0) |  | | 5 (1.3) | | 219 (13.9) | 94.7 (85.4, 98.1) |  |
| **By resident district** |  |  |  |  |  |  |  |  | |  | |  |  |  |
| Urban ^a^ | 163830 (76.5) | 170558 (79.7) | 22.0 (20.7, 23.3) |  | 81 (10.0) | 1096 (33.9) | 88.2 (84.1, 91.2) |  | | 12 (3.8) | | 313 (25.1) | 93.5 (86.5, 96.9) |  |
| Rural ^a^ | 304119 (85.1) | 308250 (86.3) | 11.8 (10.4, 13.1) |  | 84 (12.8) | 965 (36.9) | 88.8 (84.4, 91.9) |  | | 17 (6.9) | | 289 (29.5) | 89.9 (81.6, 94.4) |  |
| **By vaccination course** |  |  |  |  |  |  |  |  | |  | |  |  |  |
| Partial vaccination | 13430 (2.4) | 12245 (2.1) | 5.6 (3.1, 8.1) |  | 9 (0.6) | 67 (1.1) | 76.7 (51.7, 88.8) |  | | 2 (0.4) | | 23 (1.0) | 77.8 (4.1, 94.8) |  |
| Full vaccination | 195727 (34.3) | 199094 (34.8) | 15.9 (14.9, 16.9) |  | 92 (6.3) | 829 (14.2) | 83.4 (78.4, 87.3) |  | | 19 (3.4) | | 245 (11.0) | 86.6 (76.6, 92.3) |  |
| Booster vaccination | 258792 (45.3) | 267469 (46.8) | 17.8 (16.8, 18.8) |  | 64 (4.4) | 1165 (19.9) | 92.7 (90.1, 94.6) |  | | 8 (1.4) | | 334 (15.0) | 95.9 (91.4, 98.1) |  |
| **By vaccination intervals (weeks)** |  |  |  |  |  |  |  |  | |  | |  |  |  |
| Dose1: 0~2 | 185 (0.0) | 195 (0.0) | 17.5 (-0.9, 32.6) |  | 0 (0.0) | 1 (0.0) | NA ^d^ |  | | - | | - | - |  |
| Dose1: 3~12 | 2579 (0.5) | 2603 (0.5) | 12.8 (7.8, 17.6) |  | 2 (0.1) | 26 (0.4) | 84.9 (34.3, 96.5) |  | | 0 (0.0) | | 7 (0.3) | NA ^d^ |  |
| Dose1: 13~24 | 2138 (0.4) | 1968 (0.3) | 3.5 (-2.8, 9.3) |  | 0 (0.0) | 5 (0.1) | NA ^d^ |  | | 1 (0.2) | | 2 (0.1) | NA ^d^ |  |
| Dose1: 25~36 | 3603 (0.6) | 3035 (0.5) | -1.4 (-6.6, 3.5) |  | 2 (0.1) | 12 (0.2) | 73.0 (-37.6, 94.7) |  | | 0 (0.0) | | 3 (0.1) | NA ^d^ |  |
| Dose1: 37+ | 4854 (0.8) | 4302 (0.8) | 4.1 (-0.1, 8.1) |  | 5 (0.3) | 23 (0.4) | 66.4 (5.5, 88.0) |  | | 1 (0.2) | | 11 (0.5) | 78.3 (-73.9, 97.3) |  |
| Dose2: 0~2 | 125 (0.0) | 117 (0.0) | 7.3 (-19.3, 28) |  | - | - | - |  | | - | | - | - |  |
| Dose2: 3~12 | 5049 (0.9) | 5300 (0.9) | 15.9 (12.4, 19.2) |  | 2 (0.1) | 16 (0.3) | 78.9 (2.3, 95.4) |  | | 1 (0.2) | | 7 (0.3) | 86.1 (-34.2, 98.6) |  |
| Dose 2: 13~24 | 18901 (3.3) | 18742 (3.3) | 9.9 (7.3, 12.4) |  | 3 (0.2) | 33 (0.6) | 84.7 (47.6, 95.6) |  | | 1 (0.2) | | 12 (0.5) | 78.4 (-71.7, 97.3) |  |
| Dose 2: 25~36 | 50447 (8.8) | 49455 (8.7) | 12.2 (10.7, 13.7) |  | 27 (1.8) | 228 (3.9) | 80.8 (70.2, 87.6) |  | | 4 (0.7) | | 63 (2.8) | 86.8 (63.9, 95.1) |  |
| Dose 2: 37+ | 121197 (21.2) | 125476 (22.0) | 17.7 (16.6, 18.8) |  | 60 (4.1) | 550 (9.4) | 84.6 (78.9, 88.7) |  | | 13 (2.3) | | 163 (7.3) | 87.0 (74.7, 93.4) |  |
| Dose 3: 0~2 | 1308 (0.2) | 1261 (0.2) | 12.1 (4.9, 18.7) |  | 0 (0.0) | 2 (0.0) | NA ^d^ |  | | - | | - | - |  |
| Dose 3: 3~12 | 68340 (12.0) | 68096 (11.9) | 14.8 (13.5, 16.1) |  | 19 (1.3) | 256 (4.4) | 88.0 (80.3, 92.7) |  | | 3 (0.5) | | 73 (3.3) | 94.3 (81.3, 98.3) |  |
| Dose 3: 13~24 | 179876 (31.5) | 189466 (33.2) | 19.7 (18.6, 20.7) |  | 43 (2.9) | 893 (15.3) | 93.9 (91.3, 95.8) |  | | 5 (0.9) | | 257 (11.5) | 96.5 (91.1, 98.6) |  |
| Dose 3: 25+ | 9347 (1.6) | 8792 (1.5) | 10.1 (7.2, 12.8) |  | 2 (0.1) | 16 (0.3) | 89.4 (50.9, 97.7) |  | | 0 (0.0) | | 4 (0.2) | NA ^d^ |  |

VEs were from univariate conditional logistic regression and were not unadjusted for baseline characteristics due to the exact matching process. VEs were from univariate conditional logistic regression and were unadjusted for baseline characteristics for exact matching process. Effectiveness of at least one dose inactivated vaccines was estimated in the whole population and in subgroups defined by strata of age, gender, and residential district. Effectiveness of each vaccination course was estimated in the whole population.

a: The proportion of vaccinated individuals were calculated as the number of vaccinated people divided by the total number of people in corresponding category

b: Vaccine effectiveness for the combined age group 3-39 years.

c: Vaccine effectiveness for the combined age group 18-59 years.

d: Vaccine effectiveness in this cell had an infinite confidence interval

Figure S1. Subset 1 for inactivated vaccines estimates of effectiveness against documented SARS-CoV-2 infection, severe/critical illness and Covid-19 related death.

Neither case nor control population in subset 1 was documented to have received a COVID-19 vaccine other than an inactivated vaccine. In subset 1, each documented SARS-CoV-2 infection was matched to one test negative control (1:1 match) by age (in the same year), gender, birthplace, date of illness onset, and residential district; each severe or critical illness or Covid-19 related death was matched to four test negative controls (1:4 match) by age (in the same year), gender, birth place, date of illness onset, and residential district. The final, analytic dataset included 571,306 case-control pairs to estimate the VE for documented SARS-CoV-2 infection, 1,462 matched (a ratio of 1:4) severe/critical illness cases, and 557 matched (a ratio of 1:4) Covid-19 related deaths.

Figure S2. Subset 2 for Ad5-nCoV vaccine estimates of effectiveness against documented SARS-CoV-2 infection, severe/critical illness, and Covid-19 related death.

Neither cases nor controls in subset 2 had a history of vaccination with other than Ad5-nCoV vaccine. In subset 2, one documented SARS-CoV-2 infection was matched to one test negative control (1:1 match) by age (in the same year), gender, birth place, date of illness onset, and residential district, while one severe or critical illness, or one Covid-19 related death was matched to four test negative controls (1:4 match) by age (in the same year), gender, birthplace and residential district. The final, analytic dataset included 40,032 case-control pairs to estimate VE for documented SARS-CoV-2 infection, 547 matched (a ratio of 1:4) severe/critical illness cases, and 248 matched (a ratio of 1:4) Covid-19 related deaths.

Figure S3. The process of vaccination history retrieval.
